# Novel and unexpected bacterial diversity in an arsenic-rich ecosystem revealed by culture-dependent approaches

**DOI:** 10.1186/1745-6150-7-28

**Published:** 2012-09-10

**Authors:** François Delavat, Marie-Claire Lett, Didier Lièvremont

**Affiliations:** 1 UMR7156 Université de Strasbourg/CNRS, Génétique Moléculaire, Génomique, Microbiologie, Strasbourg, France

**Keywords:** Acid mine drainage (AMD), Alkaliphilic bacteria, Neutrophilic bacteria, Functional redundancy, Rare biosphere, Uncultured bacteria, Molecular biases, Culture-dependent approaches, *Actinobacteria*, Bacterial diversity

## Abstract

**Background:**

Acid Mine Drainages (AMDs) are extreme environments characterized by very acid conditions and heavy metal contaminations. In these ecosystems, the bacterial diversity is considered to be low. Previous culture-independent approaches performed in the AMD of Carnoulès (France) confirmed this low species richness. However, very little is known about the cultured bacteria in this ecosystem. The aims of the study were firstly to apply novel culture methods in order to access to the largest cultured bacterial diversity, and secondly to better define the robustness of the community for 3 important functions: As(III) oxidation, cellulose degradation and cobalamine biosynthesis.

**Results:**

Despite the oligotrophic and acidic conditions found in AMDs, the newly designed media covered a large range of nutrient concentrations and a pH range from 3.5 to 9.8, in order to target also non-acidophilic bacteria. These approaches generated 49 isolates representing 19 genera belonging to 4 different phyla. Importantly, overall diversity gained 16 extra genera never detected in Carnoulès. Among the 19 genera, 3 were previously uncultured, one of them being novel in databases. This strategy increased the overall diversity in the Carnoulès sediment by 70% when compared with previous culture-independent approaches, as specific phylogenetic groups (*e.g.* the subclass *Actinobacteridae* or the order *Rhizobiales*) were only detected by culture. Cobalamin auxotrophy, cellulose degradation and As(III)-oxidation are 3 crucial functions in this ecosystem, and a previous meta- and proteo-genomic work attributed each function to only one taxon. Here, we demonstrate that other members of this community can also assume these functions, thus increasing the overall community robustness.

**Conclusions:**

This work highlights that bacterial diversity in AMDs is much higher than previously envisaged, thus pointing out that the AMD system is functionally more robust than expected. The isolated bacteria may be part of the rare biosphere which remained previously undetected due to molecular biases. No matter their current ecological relevance, the exploration of the full diversity remains crucial to decipher the function and dynamic of any community. This work also underlines the importance to associate culture-dependent and -independent approaches to gain an integrative view of the community function.

**Reviewers:**

This paper was reviewed by Sándor Pongor, Eugene V. Koonin and Brett Baker (nominated by Purificacion Lopez-Garcia).

## Background

Acid Mine Drainages (AMDs) are extreme environments characterized mostly by heavy metal contaminations and very acidic conditions. It has been already shown that the presence of metals can lead to a decrease of 99.9% of the overall bacterial diversity in soil [1]. The combination of high metal concentration and acidic pH in AMDs further trigger this process, and various studies concluded on the occurrence of a low *in situ* bacterial diversity [2-4].

The Reigous is a small creek (pH 2.7-3.4) flowing down the ancient mining site of Carnoulès (Gard, France) characterized by high arsenic (up to 350 mg.l^-1^) and iron (up to 2700 mg.l^-1^) concentration in waters [5]. A follow-up study linked the progressive arsenic removal to bacterial activities, especially their As(III)-oxidizing activities [5]. In order to understand the biological processes occurring *in situ*, several studies aimed at determining the bacterial diversity in the water body or in the sediment.

In the water, both culture-dependent and culture-independent approaches had already been undertaken. The culture-independent studies pointed out the low procaryotic diversity [3,6,7], characterized by a stable composition over time. In parallel, only three bacterial genera were isolated from the water, corresponding to the genera *Thiomonas* sp., *Acidithiobacillus* sp. and *Burkholderia* sp. [8-11]. *Thiomonas* species was shown to be involved in As(III) oxidation [8,11-13] whereas the *Acidithiobacillus ferrooxidans* strain performed iron Fe(II) oxidation [10].

In the soft sediment lying below the running water, up to now only culture-independent approaches were undertaken. A recent study, combining global metagenomic, metaproteomic and RT-PCR, and deciphering both the bacterial diversity present and the community function, highlighted a low bacterial diversity in the sediment allowing the reconstruction of 7 nearly complete genomes (called CARN1 to CARN7), 5 of which representing uncultured bacteria [14]. In-depth analysis of the genomes as well as metaproteomic and RT-PCR resulted in an integrated model of the community function illustrating potential inter-species interactions. This powerful strategy has clearly enhanced the comprehension of the community function. However, in the proposed model, each of three crucial functions was each linked to only one bacterium *i.e.* the As(III) oxidation only to CARN2, cobalamin biosynthesis only to CARN1/4 (both belonging to *Candidatus* Fodinabacter communificans) or cellulose degradation only to CARN6 [14].

The aim of the present work was to investigate the bacterial diversity in the sediment by culture-dependent approaches. In a first step, we implemented various strategies to access to the largest cultured bacterial diversity. They integrated modern cultivation methods such as the use of 1) a mineral base that mimicks the AMD water conditions, 2) low organic carbon content to avoid growth inhibition of slow-growing bacteria by fast-growing bacteria, 3) less stringent growth conditions (particularly in terms of pH) to enhance bacterial growth, 4) gellan gum instead of agar as a solidifying agent, and 5) innovating techniques such as the Soil Slurry Membrane System (SSMS) [15]. These culture-dependent approaches allowed the detection of 16 genera that were never detected previously in Carnoulès, thus increasing the overall bacterial diversity by 70%. These results demonstrated that AMD bacterial diversity is larger than previously recognized. They also illustrate that culture-dependent methods remain crucial to determine the bacterial community composition and are really complementary to genomic methods to improve our understanding of natural ecosystems.

In a second step, we investigated the functional redundancy in the cultured bacterial community for important functions *i.e.* As(III) oxidation, cobalamin biosynthesis, and cellulose degradation. These results showed that by maintaining the integrity of functional processes within the bacterial community, the AMD ecosystem gains more stability and robustness than previously thought.

## Results and discussion

### Bacterial diversity in the sediment of Carnoulès by novel culture-dependent approaches

The diversity of cultured bacteria was tested in the soft and unstable sediment collected directly under the running water of Carnoulès. The physical and chemical characteristics of the running water were described elsewhere [16]. For this purpose, 11 media corresponding to commonly used media and newly designed FD media were used. The mineral base of all FD media was identical and was formulated to be as close as possible to the mineral conditions found in Carnoulès, with the exception of the absence of toxic compounds such as arsenic in order to decrease the selective pressure. The importance of the carbon concentration and the pH of the media were tested. All in all, the media used in this study varied from pH 3.5 to 9.8, and from 0.01% CAA as sole carbon source to the LB-rich medium (Table 1).

**Table 1:** Strains affiliation, isolating medium characteristics and physiological and genetic properties of the isolated strains

**Taxonomy**		**Isolation Medium**			**Metabolism and genetic**		
**Strains affiliation (isolates)**	**Closest type strain / identity (acc. num.)**	**Name**	**CAA%**	**pH**	***aioA *gene amplification**	**arsenite oxidation**	**cellulose degradation**
*Acidobacteria* bacterium	*Acidobacterium capsulatum* ATCC 51196 / 94% (NR_043386)						
**(N3B)**		FD2	0.01	5.5	-	-	-
*Bacillus* sp.	[*Brevibacterium*] *frigoritolerans* DSM 8801 / 99% (NR_042639)						
(Q9*)		LB	-	7	-	-	-
*Paenibacillus* sp.	*Paenibacillus taichungensis* BCRC 17757 / 99% (NR_044428)						
(Q8*)		LB	-	7	-	-	+
*Cellulomonas* sp.	*Cellulomonas chitinilytica* X.bu-b / 97% (NR_041511)						
(O1)		FD1	0.01	3.5			
(**E10**, J12, J13, J14, J16, K13 K5, K6, K8, L7, L8, L9, L11, L14, L15, P2, U3)		FD2	0.01	5.5	-	-	-
*Streptomyces* sp.	*Streptomyces atratus* NRRL B-16927 / 99% (NR_043490)						
**(H7)**		FD2	0.01	5.5	-	-	-
*Propionibacteriaceae*	*Luteococcus peritonei* CCUG38120 / 95% (NR_028882)						
**(H7p)**		FD2	0.01	5.5	-	-	-
*Arthrobacter* sp.	*Arthrobacter albidus* LC13 / 98% (NR_041403)						
**(J9)**		FD2	0.01	5.5	-	-	-
*Micrococcus* sp.	*Micrococcus yunnanensis* YIM / 99% (FJ214355)						
(Q7*)		LB	-	7	-	-	-
*Rhodococcus* sp.	*Rhodococcus erythropolis* N11 / 99% (NR_037024)						
**(U2)**		FD2	0.01	5.5	-	-	-
*Micromonospora* sp.	*Micromonospora coriariae* NAR0 / 99% (NR_042314)						
**(X14)**		1/100 YPD + 100 mg/l As(V)	-	8	+	-	+
*Acidocella* sp.	*Acidocella facilis* / 99% (NR_025852)						
(Q3*, Q6*)		FD1	0.01	3.5			
**(I10)**		FD2	0.01	5.5	-	-	-
(L5, Q1*, Q2*, Q4*, Q5*)		FD4	0.1	5.5			
*Acidisoma* sp.	*Acidisoma tundrae* WM1 / 98% (NR_042705)						
(**K16**, L2)		FD1	0.01	3.5	-	-	+
*Methylorosula* sp.	*Methylorosula polaris* V-22 / 98% (EU586035)						
**(N4)**		FD2	0.01	5.5	-	-	-
*Ancylobacter* sp.	*Ancylobacter dichloromethanicus* DM16 / 97% (EU589386)						
**(X1)**		CDM	-	7	-	-	-
*Pseudomonas* sp.	*Pseudomonas psychrotolerans* C36 / 99% (NR_042191)						
(**K7**, L10)		FD2	0.01	5.5	-	-	-
*Rhodanobacter* sp.	*Rhodanobacter ginsengisoli* GR17-7 / 99% (NR_044127)						
(**L12**, N3J, U4, U5, U7)		FD2	0.01	5.5	-	-	-
*Dyella* sp.	*Dyella japonica* XD53 / 97% (NR_040974)						
(**K4**)		FD2	0.01	5.5	-	-	-
*Xanthomonadaceae*	*Dokdonella koreensis* DS-123 / 93% (NR_043322)						
(**X11**)		mm126	-	5	-	-	-
*Thiomonas* sp.	*Thiomonas cuprina* NBRC 102145 / 98% (NR_041628)						
**(X19)**		1/100 YPD + 100 mg/l As(III)	-	9.8	+	+	ND

In brackets are the strains code and * indicates the strains isolated via SSMS. In bold are the isolates used for 16S rRNA gene sequencing and physiological experiments. Accession numbers are as follows: N3B (FR874231); Q9 (FR874233); Q8 (FR874235); E10 (FR874226); H7 (FR874234); H7p (FR874241); J9 (FR874236); Q7 (FR874239); U2 (FR874228); X14 (FR874224); I10 (FR874225); K16 (FR874240); N4 (FR874230); X1 (FR874229); K7 (FR874238); L12 (FR874232); K4 (FR874237); X11 (FR874227) and X19 (FR874242). ND: Not determined.

**Figure 1:**
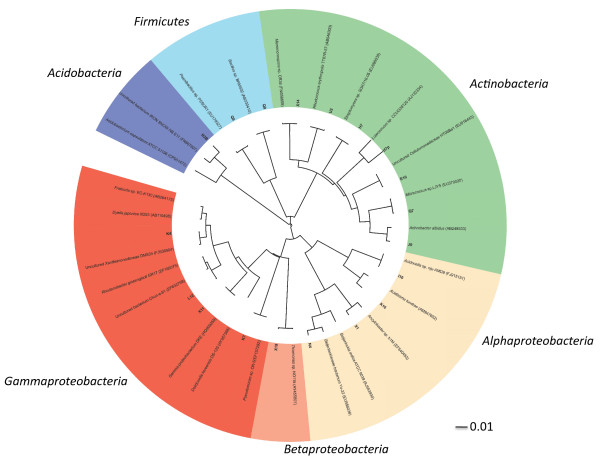
**Phylogenetic tree representing the taxonomic affiliation of the Carnoulès isolates.** The 16S rRNA gene sequences of the isolates (in bold) and their closest relatives were aligned with the MEGA 5 implementation of ClustalW algorithm. Neighbor joining tree was performed with this software and tree was drawn up using the website ITOL (http://itol.embl.de/).

The different media and growth conditions allowed the isolation of 49 bacterial strains. All strains were identified by comparison of their nearly complete 16S rRNA gene sequences with the NCBI-nr and RDP databases (Table 1). The isolates were assigned to 19 genera belonging to 4 phyla (Figure 1). Among the 19 genera detected, 9 (47%) were found within *Proteobacteria*, 7 (37%) within *Actinobacteria*, 2 (11%) within *Firmicutes* and 1 (5%) within the phylum *Acidobacteria*. Among the *Proteobacteria*, members of the α- (4 out of 9 genera, 44.5%) and γ-subdivisions (4 out of 9, 44.5%) were well represented, while only one member of the β-subdivision (1 out of 9, 11%) was isolated. *Actinobacteria* were all found within the order *Actinomycetales* and were assigned into 5 suborders, namely *Micrococcineae* (3 genera out of 7, 42.8%), *Propionibacterineae* (1 out of 7, 14.3%), *Corynebacterineae* (1 out of 7, 14.3%), *Streptomycineae* (1 out of 7, 14.3%) and *Micromonosporineae* (1 out of 7, 14.3%). The 2 *Firmicutes* were found within the order *Bacillales* whereas the *Acidobacteria* was member of the subdivision 1 of this phylum.

Interestingly, no growth was detected on FD3, FD5 and FD6 characterized by very low pH (FD3) and/or high CAA concentration (respectively FD5 and FD6). By contrast, FD2, characterized by less acidic pH (5.5) and low carbon concentration (0.01% CAA), allowed the isolation of the largest diversity, with strains belonging to 11 out of the 19 genera. Those genera were found within *Proteobacteria* (5 genera out of 11, 45%), *Actinobacteria* (5 genera out of 11, 45%) and *Acidobacteria* (1 genera out of 11, 9%). It should be noted that FD2 was the most appropriate medium to isolate *Actinobacteria*, since it allowed the isolation of 5 bacterial strains out of the 7 strains affiliated to phylum *Actinobacteria*. FD1 medium, differing from FD2 in the pH used (3.5 for FD1) allowed the isolation of representatives of 3 genera, namely *Acidisoma, Acidocella* and *Cellulomonas.* The latter two were however also isolated on FD4 and/or FD2 media. Thus, the isolates K16 and L2, both belonging to genus *Acidisoma,* were the only bacteria that were isolated solely on very acidic medium. These results indicate that the increase of the incubation time and the reduction of the carbon concentration allowed the isolation of the slow-growing bacteria, as previously suggested by Vieira-Silva [17]. It also shows that the complete mimicking of *in situ* conditions in terms of pH (3.5) resulted in a poor recovery of genera, since only *Acidocella, Acidosoma*, and *Cellulomonas* have been isolated on FD1 (Table 1). These results are consistent with those of Hallberg and Johnson [18] who isolated moderate acidophilic bacteria by increasing the pH of the growth medium compared to the natural environment as well as with data reported by Hao [19] who detected neutrophilic bacteria in AMD.

Another important factor to explain this diversity is the use of gellan gum instead of agar as a solidifying agent. Indeed, when grown on their culturing medium with agar instead of gellan, the growth rate of all strains was slower, except for strains affiliated to the genera *Arthrobacter* (J9), *Acidocella* (L5, Q1, Q2, Q3, Q4, Q5, Q6 and I10), and *Acidisoma* (K16, L2) (data not shown). N3B and H7p, affiliated to the phylum *Acidobacteria* and the family *Propionibacteriaceae*, respectively, were even unable to grow on agar plates. These results are consistent with a previous work showing that the cultured microbial diversity was increased with gellan gum when compared with agar [20].

Direct spreading on modified m126 medium (mm126), CDM and 1/100 YPD media supplemented with 100 mg.l^-1^ As(III) or As(V) allowed the isolation on each medium of only one bacterial genus, respectively a new genus of the family *Xanthomonadaceae* (X11), *Ancylobacter* sp. (X1), *Thiomonas* sp. (X19) and *Micromonospora* sp. (X14) (Table 1). We showed here that when using a broad range of pH (from 3.5 to 9.8), neutrophilic and even alkaliphilic bacteria could be isolated (Table 1). Especially, using a high pH medium (9.8), we succeeded in isolating a strain belonging to the genus *Thiomonas* sp. (X19). Several bacteria belonging to the group 1 of *Thiomonas* had been previously isolated from the water of Carnoulès [8,9,11]. However, significant differences between the 16S rRNA sequence of X19 and other *Thiomonas* from group 1 (*e.g.* 90% identity between X19 and *Thiomonas* sp. CB2 (FJ014922) over the full alignment) showed that X19 does not belong to the group 1 of the *Thiomonas* genus. By contrast, X19 and CARN2 (one of the dominant species detected previously by a culture-independent approach [14]) differed by only a single nucleotide mismatch over the nearly full length of the 16S rRNA gene. Thus, X19 corresponds to the first representative of the group 2 of the *Thiomonas* isolated in Carnoulès so far. Members of this genus are routinely grown on m126 medium (pH 5) [12,13] and were not known to grow in alkaline conditions [21], to the contrary to X19. Nevertheless, spreading X19 on this modified mm126 medium then led to the formation of visible colonies after 10 to 14 days as compared to the 14 to 21 days needed on 1/100 YPD + 100 mg.l^-1^ As(III) plates. When compared to other *Thiomonas* bacteria, X19 grew however much slower on mm126 (data not shown) [12,13]. Since the CARN2-like X19 strain adapts *in vitro* to different conditions such as high pH variation, one can hypothesize about its adaptation potentialities to various *in situ* physico-chemical conditions.

Lastly, a 10 days-incubation of the samples on SSMS [15] followed by the spreading of the microcolonies on solid media [22] allowed the culture of isolates belonging to 4 genera. Among them, 3 were detected only on LB plates. Those strains belong to the genera *Bacillus* (Q9) and *Paenibacillus* (Q8) from the phylum *Firmicutes* and *Micrococcus* (Q7) from the phylum *Actinobacteria*. The advantage of this strategy was here to avoid fungal contamination compared to direct spreading for which different moulds invaded the LB plates, despite the presence of antifungal agents. *Acidocella* (Q1 to Q6) were also detected after growth on SSMS and spreading on FD1 media. They shared an identical 16S rRNA gene sequence with strains isolated directly on FD media.

Our results indicate that the isolated strains are highly specific to the medium used for primary isolation, since the majority of them were found on only one medium. As previously mentioned, exceptions are found for members of the genus *Cellulomonas* sp. which were isolated on both FD1 and FD2 media and *Acidocella* sp. which were found on FD1, FD2 and FD4 media.

### Isolation of previously uncultured bacteria

The culture strategy led here to the isolation of representatives of 3 new genera as defined by Tindall *et al*. [23]. Indeed, these 3 isolates shared less than 95% 16S rRNA gene sequence identity with their closest taxonomically characterized species. The first one (N3B) belongs to the phylum *Acidobacteria* and shared 98% 16S rRNA gene sequence identity with uncultured clone IRON_SNOW_NB_E11 (FR667807) whereas its closest taxonomically characterized species was *Acidobacterium capsulatum* (CP001472) with 94% identity. *Acidobacteria* correspond to some of the most abundant microorganisms in the environment but are recalcitrant to cultivate in laboratory [24]. Only 8 genera from this phylum have been taxonomically isolated so far [25]. The recent metagenomic investigation of Carnoulès AMD, led to the detection of a strain of *Acidobacteria i.e.* CARN3 [14]. However, N3B showed less than 95% 16S identity with CARN3. The second one (X11) belongs to the family *Xanthomonadaceae*. X11 sequence was closely related to the uncultured *γ-Proteobacterium* DKE (100% identity, HQ909259) but the closest taxonomically characterized strain was *Dokdonella koreensis* (AY987368) with less than 94% identity. X11 and its closest related strains are common inhabitants of mines and acidic environments, since BLAST analysis revealed the presence of their 16S rDNA in Carnoulès (clone CG-36 (FN391831) [14]) as well as in various other acid mine drainages and acidic waters (*e.g.* “Rio Tinto” in Spain, “Lower Red Eyes” in Pennsylvania, “Wheal Jane” in England).

The third one is H7p, a member of the *Propionibacteriaceae* family. Its best matching 16S rRNA gene sequence is *Luteococcus* sp. (AJ132334) found in human peritoneum sharing only 95% identity with H7p. No better identity was found in any database (NCBI-nr and RDP) even with «uncultured bacteria ». The closest taxonomically characterized strain is *Luteococcus peritonei* (NR_028882) also sharing 95% identity with H7p. It should be noted that the species *Propionibacterium acnes*, belonging to this family but presenting only 90% sequence identity with H7p, has been previously detected in AMDs [18]. All these results indicate that H7p belongs to a novel genus that has never been detected previously in AMDs or in any other environment.

### From the bacterial diversity to the community function

Deciphering the biological processes occurring *in situ* in any environment requires both the knowledge of the overall bacterial diversity and the comprehension of the role of each microorganism in the community function. However, the more diverse is the community, the more difficult it is to understand the role of each taxon in this community. In this sense, acid mine drainages are well-suited models as the bacterial community is considered to harbour a low diversity, with only a few dominant taxa [2-4]. In the AMD of Carnoulès, both culture-dependent and culture-independent approaches were already undertaken to unravel this diversity when using the water body as template [3,6-11]. However, only culture-independent approaches were carried out when searching in the soft sediment collected directly under the running water [14,26]. All these experiments highlighted the low bacterial diversity occurring both in the sediment and in the above running water.

**Figure 2:**
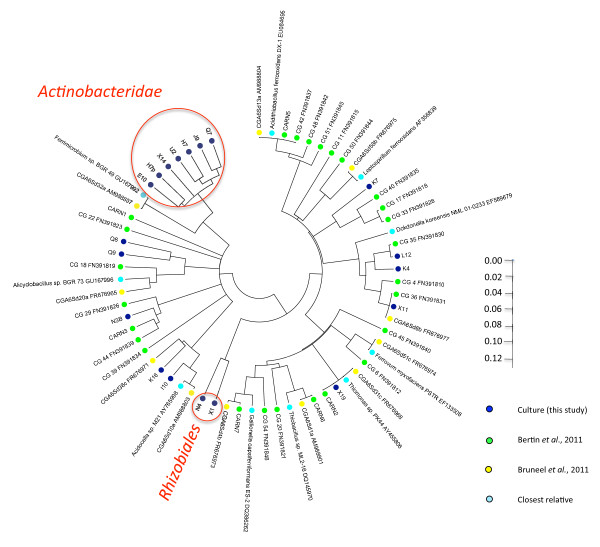
**Phylogenetic tree representing all taxa detected in the sediments of Carnoulès.** The 16S rRNA gene sequences of the isolates (filled dark-blue circles), closest relative (filled light-blue circles) and other taxa found in Carnoulès by Bertin *et al.*, 2011 (filled green circles); Bruneel *et al.,* 2011 (filled yellow circles) were aligned with the MEGA 5 implementation of ClustalW algorithm. Neighbor joining tree and tree representation were performed with this software. The open red circles correspond to phylogenetic groups detected only by culture-dependent approach (this study).

In the present study, we succeeded in isolating 49 strains belonging to 19 genera. Importantly, 16 of these genera had never been detected previously on this site [14,26], thus increasing the overall bacterial diversity in the sediments by 70% (Figure 2). Indeed, only 3 strains, belonging to *RhodanobacterThiomonas* and the new genus belonging to the family *Xanthomonadaceae* (X11) were found both by our culture-dependent investigations and by culture-independent approaches [14,26]. Moreover, members of 3 phyla were detected only by culture-independent approaches, namely *SpirochaetesNitrospira* and the novel phylum represented by CARN1/CARN4 [14]. On the other hand, phylogenetic groups like the subclass *Actinobacteridae* or the order *Rhizobiales* were only detected via the present culture strategy. A phylogenetic tree representing all taxa detected in the sediments of Carnoulès by both methods allowed to highlight the overall microbial diversity and to point out the complementarity between the approaches (Figure 2).

To confirm this result, specific primers ( Additional file 1) targeting each genus previously undetected by previous culture-independent studies [14,26] were designed and tested by using metagenomic DNA from the Carnoulès sediment as template. Among them, only H7p, member of the new genus within *Propionibacteriaceae*, had an identical sequence in the metagenomic DNA. This result suggests that only this latter strain could have been detected by the previous molecular techniques, if more clones would have been sequenced. Indeed, no rarefaction curve was presented in the previous studies. More generally, this result suggests that the DNA concentration corresponding to all other cultured strains was too low or even absent to be detected inside the metagenome mixture. Therefore, one could hypothesize that the isolated strains correspond to rare populations [27] in the sediment of Carnoulès. Indeed, rare bacteria should be considered [28] since recent studies indicated that even if they are present at a dormant or a spore stage, they may become active and abundant when the *in situ* conditions change [29,30]. Moreover, it has been shown that rare bacteria eventually not detected by molecular techniques can significantly contribute to the global functioning of any ecosystem [31,32]. It is also recognized that culture may be a powerful strategy to access to this previously undetected rare biosphere [33,34]. Here, the dilutions used (100 μl of the dilutions 10^-1^ and 10^-2^ were spread on each plate) allowed to estimate the population of each genus by several hundreds to thousands CFU per gram of sediment. Alternatively, they can have been missed out previously because their DNA was inaccessible by the extraction methods used for metagenomic investigations. Indeed, it is already known that *Actinobacteria*, representing 37% of the genera detected here, are often underestimated by molecular approaches due to poor DNA extraction [35,36]. For instance, it has been proved that *Micromonospora* species (as X14) are insensitive to most lysis treatments [37]. More generally, the metagenomic DNA protocol affects importantly the DNA recovered [38] and thus the bacterial diversity which is detected.

No matter the current *in situ* ecological relevance of the strains, they can play an essential role when the physico-chemical conditions change. In this sense, it remains crucial to approach as far as possible the full bacterial diversity to better understand how a community works and evolves. In addition to the extension of the bacterial diversity, our work also allows to test some physiological characteristics and to provide potential role(s) of the strains for the community function. As such, we decided to screen for functions, which are crucial for the survival of the bacterial community in AMDs but which lack the necessary redundancy, *i.e.* functions that are carried only once in the previous global metagenomic approach [14].

The first tested function concerns the biosynthesis of cobalamin (vitamin B12). Interestingly, all FD media were designed without cobalamin and vitamin-free CAA was used. Thus, all bacteria growing on such media are prototroph for cobalamin. Indeed, 12 genera out of the 19 (belonging to *Acidobacteria* bacterium*, Cellulomonas, Streptomyces, Propionibacteriaceae* bacterium*, Arthrobacter, Rhodococcus, Acidocella, Acidisoma, Methylorosula, Pseudomonas, Rhodanobacter* and *Dyella*, see Table 1) were isolated on the newly designed FD media. The cobalamin biosynthesis pathway genes were previously found by metagenomics only in CARN1/CARN4 (both grouped within the uncultured bacterium *Candidatus* Fodinabacter communificans) whereas the other genomes (such as CARN2 and CARN5) carry the cobalamin transporter *btuC* gene. Moreover, the photosynthetic microorganism *Euglena mutabilis* isolated from Carnoulès was recently shown to be auxotroph for cobalamin [39]. *Candidatus* Fodinabacter communificans was therefore thought to be essential for the community, at least by providing vitamin B12 for the rest of the community [14]. It is tempting to hypothesize that some of the isolated strains in the present work are able to produce this vitamin and provide it to the rest of the AMD community.

The second function tested was the cellulose degradation. As the AMD of Carnoulès is at least partly oligotroph [14], any possibility to catabolize unusual nutrients would be advantageous for the corresponding bacterium [40]. The released by-products can be also useful for the rest of the community as it can allow syntrophic interactions. As such, the metagenomic approach allowed the detection of the genes encoding proteins responsible for the degradation of the cellulose polymers only in the genome of CARN6 [14]. The ability to degrade the cellulose was tested for one representative of each of the 19 genera (strains code E10, H7, H7p, I10, J9, K4, K7, K16, L12, N3B, N4, Q7, Q8, Q9, U2, X1, X11, X14, X19), using carboxymethylcellulose (CMC) as substrate. A yellow halo was observed for K16 (*Acidisoma* sp.), X14 (*Micromonospora* sp.) and Q8 (*Paenibacillus* sp.), demonstrating their ability to degrade this complex compound (Table 1). When used with the strain X19 on mm126 plates, the degradation test resulted in a coagulation of the Congo red dye, which turned violet, making impossible the lecture of the results. This reaction, due to the acidification of the medium, has already been described [41]. To our knowledge, this is the first time that polymer-degrading activities of bacteria isolated from oligotrophic AMDs were physiologically demonstrated. Q8 was then further studied in details for its numerous polymer-degradation activities under a wide range of stress conditions *i.e.* for its ecological relevance when ambient environmental conditions change [16].

The third function is As(III) oxidation, an important function in Carnoulès since it allows the co-precipitation of arsenic and iron and leads to a sharp decrease of the arsenic concentration in the AMD [5] and to the detoxification of the ecosystem. We tested in laboratory conditions the As(III) oxidation capability for one representative of each genus (strains code E10, H7, H7p, I10, J9, K4, K7, K16, L12, N3B, N4, Q7, Q8, Q9, U2, X1, X11, X14, X19) in their liquid culturing medium supplemented with 100 mg.l^-1^ As(III). All strains were able to grow but only X19 was able *in vitro* to oxidize As(III) to As(V) as measured by HPLC-ICP-OES experiments (Table 1). The isolation of the CARN2-like X19 strain is of importance, since it allowed to test and measure physiologically its As(III)-oxidizing potentiality previously hypothesized by metagenomic and metaproteomic [14]. This strategy allowed therefore to confirm one major role for CARN2 in the community function. In accordance to the 16S rRNA gene sequence similarity between X19 and CARN2, the *aioA* gene sequence (984 bp) of X19 encoding the large subunit of the arsenite oxidase amplified with degenerated primers [42] shared 98% identity with one copy of the *aioA* gene from CARN2 (CARN2_1330).

It should be noted that we obtained a specific sequence of the *aioA* gene from X14, belonging to the phylum *Actinobacteria*. Despite the absence of oxidation measured in laboratory conditions, X14 may therefore be able to also oxidize As(III) *in situ* as does CARN2. Interestingly, the X14 *aioA* sequence was 100% identical over its full length (989 bp) to the *aioA* gene from *Thiomonas* sp. CB2 (EU339212), belonging to the phylum *Proteobacteria* and previously isolated from the Carnoulès water [8]. This observation suggests a recent horizontal gene transfer (HGT) between these 2 bacteria, belonging to very distant phylogenetic groups. The occurrence of HGT for the *aioA* gene had previously been observed in another study site [43] but never in Carnoulès, further highlighting the complex interactions between bacteria *in situ*.

## Conclusions

Our study provided evidences that culture-dependent approaches enable the characterization of a different diversity compared to the one obtained by culture-independent approaches, highlighting the complementarity between the 2 approaches. We also pointed out that the community structure is not as simple as previously established (a 70% increase in overall diversity). Functional experiments showed that important community functions, such as cobalamin biosynthesis, the degradation of cellulose and the oxidation of As(III) are redundant in the ecosystem thus increasing the functional robustness essential for any ecosystem. Additionally, the isolation of neutrophilic or even alkaliphilic strains further highlights the capability carried by the whole community to adapt to *in situ* conditions changes such as an increase of the pH, thus improving the knowledge of the system resilience. We showed that it remains crucial to associate culture-dependent and culture-independent approaches to gain an integrative view of the community structure and function.

However, measuring the exact *in situ* role of each non-dominant species remains hard to determine, since they are hardly detectable with standard molecular techniques. Specific FISH-probes experiments can be performed more easily after isolation, but the relative abundances remains unknown, since no (or too few) DNA was recovered from the isolated genera. The determination of the full extent of the microbial diversity is therefore still challenging, and considerable efforts in terms of technologies and work have to be undertaken to approach this aim.

## Methods

### Soil sample and preparation

Sediment samples (up to 2 cm deep) were collected in November 2009 from the soft and unstable sediment of the Acid Mine Drainage (AMD) located in Carnoulès, Gard (France), at the station called CowG (44°07'01.80''N/4°00'06.90''E) [6]. The samples were then stored at 4°C in sterile 50 ml tubes until use.

Sediment sample was serially diluted with filtered-sterilized Carnoulès water and 100 μl of the dilutions 10^-1^ and 10^-2^ were plated on solid media. All colonies were then isolated by streaking at least three times to ensure purity.

### Media used in this study

Unless otherwise stated, all chemicals and reagents were supplied by Sigma-Aldrich.

Culture media used for bacterial growth were as follows:

LB medium (MP Biomedicals); m126 described in [13] without Na_2_HPO_4_ (hereafter called mm126); CDM described in [44]; a one hundred-fold dilution of YPD (MP Biomedicals) medium supplemented with 100 mg.l^-1^ As(III) or 100 mg.l^-1^ As(V).

Additionally, new synthetic media were designed, composed of (per litre MQ-water) 1.5 g KH_2_PO_4_ (VWR); 10 ml of a 35 g.l^-1^ CaCl_2_.2 H_2_0 (Fisher Scientific) solution; 10 ml of a 20 g.l^-1^ MgSO_4_.7 H_2_0 (Euromedex) solution; 10 ml of a 30 g.l^-1^ (NH_4_)_2_SO_4_ solution; 10 ml trace solution (contains: 30 mg.l^-1^ CuSO_4_.5 H_2_0; 1.5 g.l^-1^ MnSO_4_; 3 g.l^-1^ ZnSO_4_.7 H_2_0; 34 mg.l^-1^ CoCl_2_.6 H_2_0) and 10 ml of a vitamin solution (per litre): 100 mg riboflavin, 100 mg thiamine, 60 mg pyridoxine, 2 mg folic acid, 0.25 mg lipoic acid. This mineral base was completed with either 1%, 0.1% or 0.01% final concentrations of vitamin-free casaminoacids (CAA) (Fisher Scientific). Those media were then adjusted with H_2_SO_4_ and KOH either to pH 3.5 or 5.5, making 6 media (hereafter called FD1 (0.01% CAA/pH3.5); FD2 (0.01% CAA/pH5.5); FD3 (0.1% CAA/pH3.5); FD4 (0.1% CAA/pH5.5); FD5 (1% CAA/pH3.5) and FD6 (1% CAA/pH5.5), see Table 1). The vitamin solution was sterilized by filtration and added to the autoclaved medium. It should be noted that the FD media precipitate above 5.5 and thus should be prepared with caution. For solid cultures, 1.5% gellan gum (Sigma) was used as a solidifying agent.

After incubation, bacterial colonies were isolated and re-streaked on the same FD media, with yeast extract (100 mg.l^-1^) instead of the vitamin solution (culturing medium). *Pseudomonas* and *Rhodococcus* strains were routinely cultured on LB plates and *Thiomonas* on mm126 plates.

In addition to those standard media, microcultivation in a soil slurry membrane system (SSMS) was used as described by Ferrari *et al*. [15]. Briefly, sediment sample taken from the same site was used as a growth medium in an inverted Tissue Culture Insert (TCI), and one millilitre of a 1:100 dilution sample was filtered on a Polycarbonate Membrane (PCM). This PCM was placed onto the inverted TCI, which supplied nutrients for the bacteria fixed on the PCM. After the incubation time (10 days at 20°C), the membrane was removed, cut with a sterile razor blade and vortexed one minute with 1 ml 0.9% NaCl and 100 μl of the supernatant was then spread onto LB and all FD media.

Incubation on mm126, CDM, 1/100 YPD + 100 mg.l^-1^ As(III) or As(V) were done at 25°C; LB at 30°C and FD media at 20°C for up to 4 weeks.

For long-term storage, all strains were stored at −80°C in 20% glycerol.

### Cellulose degradation

Cellulolytic activity was detected on all culturing media supplemented with 0.2% carboxymethylcelullose CMC (Sigma). After the incubation time, colonies were stained with Congo red (0.2%) for 20 minutes and plates were washed with 1 M NaCl. Cellulase-expressing colonies were surrounding by a yellow halo against a red background.

### Molecular and *in silico* analyses

DNAs were extracted using the Wizard Genomic DNA purification kit (Promega) according to the manufacturer’s protocol. 16S rRNA genes were amplified using fD1 and rD1 primers [45]. PCR products were sequenced (Millegen, France) and DNA sequences were analyzed using the NCBI-nr BLAST program and the RDP database. All the sequences obtained were submitted to the EMBL databases under accession numbers FR874224 to FR874242.

Sequences were aligned with the MEGA 5 implementation of ClustalW algorithm (http://www.megasoftware.net/) and 5^′^ and 3^′^ extension were trimmed. Neighbor joining phylogenetic trees were performed with this software and trees were drawn up using the iTOL website (http://itol.embl.de/) if necessary.

Primers aoxBM1-2F (5^′^-CCACTTCTGCATCGTGGGNTGYGGNTA-3^′^ and aoxBM3-2R (5^′^-TGTCGTTGCCCCAGATGADNCCYTTYTC-3^′^) were used to amplify the partial sequence of the *aioA* gene (previously named *aoxB/aroA*/*asoA *[46] of one representative of each genus, as defined by [42]. Amplicons were sequenced and DNA sequences were analyzed using the NCBI-nr BLAST program. The sequences obtained for X14 and X19 were submitted to the EMBL databases under the accession numbers HE588125 and FR874243, respectively.

Specific primers targeting the 16S rRNA gene of each genus newly detected by cultivation approaches were designed ( Additional file 1). Forward primers corresponded to the V2 hypervariable region (*Escherichia coli* positions 137–242) and reverse primers corresponded to the V5 hypervariable region (*E. coli* positions 822–879). Because K4 and L12 showed exact identity in the V5 region, the reverse primers for these strains corresponded to the V7 hypervariable region (*E. coli* positions 1117–1173). Amplification using the metagenomic DNA from the Carnoulès sediment as template was performed with the following conditions: 35 amplification cycles of 95°C for 10 min, 58°C for 1 min 20 s, 72°C for 1 min 30 sec, followed by a final elongation cycle (72°C for 10 min).

### Arsenic speciation

One representative of each genus was tested for As(III) oxidation. Their corresponding culturing media were supplemented with 100 mg.l^-1^ As(III), adjusted to pH 5.5 and autoclaved. Media without inoculation was used as a control for abiotic oxidation. After the incubation time, the supernatant were filtered and 10-fold diluted with sterile Milli-Q system (Millipore) water and arsenic speciation was performed by HPLC-ICP-OES. Separation was performed on a reversed-phase polymeric resin (Hamilton, PRP-X100, 250 mm × 4.1 mm *i.d.*, particle size 10 μm) equipped with the corresponding guard column. Arsenic compounds were eluted with a phosphate buffer [44]. For ICP-OES experiments, a Varian 720 ES operating at a forward power of 1.2 kW and equipped with a Meinhard type nebulizer was used. Flow-rates: Plasma gas = 15 l.min^-1^, nebulizer gas = 1 l.min^-1^ (optimized each day), auxiliary gas = 1 l.min^-1^. Wavelength was fixed at 193.7 nm. All experiments were done at least in duplicates.

## Competing interests

The authors declare that they have no competing interests.

## Authors’ contributions

FD, MCL and DL conceived, supervised and coordinated this study. FD carried out all experiments and interpretation of data. FD wrote the manuscript and MCL and DL critically revised the manuscript. All authors read and approved the final manuscript.

## **Reviewers’ comments**

Reviewer's report

**Reviewer number:** 1

Referee 1 : Prof. Sandor Pongor, International Centre for Genetic Engineering and biotechnology, Trieste, Italy

Report form:

Multispecies microbial communities are a major form of life so it is crucially important to understand the factors underlying their stability. Metagenomic approaches are playing an increasingly important role in studying complex microbial communities and the research community is often led to believe that bacterial communities are what we see through culture-free metagenomic analysis. Delavat and associates have analyzed the microbial community of an arsenic rich Acid Mine Drainage (AMD) site in Carnoulès (France), a site which was believed to be of low biodiversity, based on previous metagenomic studies. Delavat and associates showed, using specific culture media, that the number of species is 70% higher than previously thought and the community includes new phylogenetic groups, such as the subclass *Actinobacteridae* or the order *Rhizobiales* which were not detected by culture-free methods. While the work is well presented and adequately documented, and shows very well the difference that exists between cultures and culture free methods in this particular case, there are a number of subjects that the authors may want to expand.

First, what is the main interest of the Carnoulès site as compared to other AMDs, and how do the present results compare to data (especially the comparison of cultured vs. non-cultured approaches) obtained on other AMD sites.

***Authors’ response:****Carnoulès is an interesting study site, essentially because this site is monitored since more than 15 years (first paper published in 1996 *[47]*, 25 papers since according to Scopus). Chemists, microbiologists, mineralogists work together to elucidate how the whole system functions. Physico-chemical characteristics of sediments and waters are monitored since years and the question of the role of bacteria in the natural attenuation occurring in situ has been the subject of numerous studies. A second point concerns the origin of this AMD, which does not only result of the action of rainwater. The Reigous spring is located below the sterile mining residues, and drains away through them. This dynamic system is maintained throughout the year.*

*To answer to the second part of the question, we put forward that most of the studies focused on the bacterial species richness in AMDs relied on culture-independent approaches carried out on the AMD water. They have all described a bacterial ecosystem with few species. Moreover, the bacterial species detected in these different studies, carried out over the years and all year round (winter, summer…) were similar, strongly suggesting a temporal stability of the bacterial community composition. However, it is true that we cannot rule out any partial variations over time, especially within the rare biosphere *[48]*.*

Thus, our study focused on the AMD sediment, and implemented only culture-dependent approaches giving us the opportunity to explore differently an AMD.

*The comment concerning the comparison of our data with those obtained in other AMDs lead to mixed answers: unfortunately there is, to our knowledge, only three studies*[4,49,50]*comparing the bacterial diversity obtained by culture and by molecular approaches in AMDs. Nevertheless, they all used only one isolating medium and did not aim at determining the largest cultured bacterial diversity. Indeed, AMDs are often either 1) screened for the bacterial diversity through molecular approaches *[3,51,52]*or 2) screened by culture-dependent approaches for targeted bacteria, such as iron- or arsenic-oxidizing bacteria *[8,18]*. Thus, AMDs were never investigated in order to determine the largest cultured bacterial diversity.*

Second, what are spatiotemporal variations of the community composition, can one expect a change in species composition with respect to space and, for instance, the time of the year.

***Authors’ response:****See the above comment for the answer.*

**Quality of written English:** Needs some language corrections before being published

***Authors’ response:****The manuscript has been completely revised for corrections.*

The authors would like to thank Reviewer 1 for his comments.

**Reviewer number:** 2

Referee 2 : Dr. Eugene Koonin, NCBI, NLM, NIH, United States of America

Report form:

The findings described here are of interest - in my opinion, mostly from the methodological standpoint, demonstrating the complementarity of culture-independent and targeted culturing approaches in the characterization of a microbiota. This seems to be becoming a common theme in modern microbiological studies. It is unclear to me in what sense the results described here are unexpected as claimed in the title. I think added microbial diversity detected by culturing is exactly what one should expect which does not make the findings unimportant.

***Authors’ response:****The term “unexpected” was used because, as Reviewer 1 pointed out “the research community is often led to believe that bacterial communities are what we see through culture-free metagenomic analysis”. In this sense, the detection of bacteria previously undetected in numerous culture-independent studies is not expected. It is however true that one could expect that the microbial diversity detected by both approaches would be different. But, studies aiming at comparing culture-independent and –dependent approaches often led to the conclusion that the bacterial diversity detected by molecular approaches is much higher than the one detected by culture *[53,54]. *Our results provide evidence of a 70% increase of the overall bacterial diversity thanks to culture-dependent approaches. These reasons led us to use the word “unexpected”.*

Also, there is some language in the manuscript that I find irrelevant. For instance (from the Conclusion section of the Abstract): “The isolated bacteria may be part of the biosphere which remained previously undetected due to molecular biases”. Obviously, the newly isolated bacteria have not been detected previously, obviously, they are part of the biota (not biosphere). What is the specific meaning of this sentence?

***Authors’ response:****This has been corrected in the manuscript, by adding the missing word “rare” before “biosphere”. Indeed, the bacteria detected in this study may be part of the “rare biosphere” as defined by Sogin et al. *[27]. *Members of this rare biosphere can remained undetected because of molecular biases exposed in the manuscript.*

**Quality of written English:** Needs some language corrections before being published.

***Authors’ response:****The manuscript has been completely revised for corrections.*

The authors would like to thank Reviewer 2 for his comments and for the interest expressed for the study. We fully agree with him to say that coming back to culture is becoming a common theme, and a complement to molecular approaches.

Reviewer number: 3

Referee 3 : Dr. Brett Baker (nominated by Dr. Purificacion Lopez-Garcia), Department of Earth and Environmental Sciences, University of Michigan, Ann Arbor, MI, USA.

Report form:

Delavat et al. employed culturing-based approach in an attempt to extend our knowledge of diversity and metabolisms of an AMD impacted creek with a pH range of 2.7-3.4. Previous studies of this creek and other AMD sites (Iron Mountain being the most extensively studied) have shown that it is low in species richness, not “low biodiversity” as the authors state.

***Authors’ response:****Indeed, even if the term “biodiversity” is frequently used for bacterial diversity (for review, see *[55]*), “species richness” is more appropriate and is used in the revised manuscript.*

I have several issues with the studies experimental design and the conclusions drawn from the results. If the pH of the creek is 2.7-3.4 why would you use media that ranges from 3.5-9.8, which is not even in the range of the creek? 17 of the 23 isolates listed in Table 1 were obtained on media with pH 5.5 and up.

***Authors’ response:****The preparation of media covering a large range of pH should allow the isolation of non-acidophilic bacteria or even alkaliphilic ones, that could not grow on acidic media. That is exactly what we obtained, since the majority of them were not isolated at pH 3.5 (close to the in situ pH), but at higher pH. Media such as R2A (pH 7.0) are frequently used for the isolation of bacteria, even in acidic environments. For example, this medium was used to isolate Thiomonas strains *[8]*which 1) are known to play an important role in the community function of Carnoulès, by oxidizing As(III) and 2) are not extreme acidophilic bacteria *[56]*. Moreover, we assume that some bacteria isolated here may not currently play important roles in situ. However, they can act as a “seed bank”, becoming important when the environmental conditions change *[57]*.*

The authors state that this was an attempt to uncover diversity not previously seen in AMD. However, proving that these are truly members of the creek would require more evidence which could be provided in the form of FISH analyses of creek samples. That being said I don’t believe that most of the isolates are endemic to the site being studied. *Bacillus, Arthrobacter, Micromonospora, Pseudomonas*, etc. are common contaminants and are surely ubiquitous to laboratories.

***Authors’ response:****We agree with Reviewer 3. Bacillus, Arthrobacter, Micromonospora and Pseudomonas genera are common contaminants. Nevertheless, they were also previously detected in the Rio Tinto *[58]*or in arsenic-contaminated soils *[59]*. Moreover, the genome of Micromonospora sp. X14 contains a fragment of the aioA gene sharing 100% nucleotidic similarity with the aioA gene of Thiomonas sp. CB2 isolated previously from Carnoulès. This indicates a horizontal gene transfer that occurred in situ. Thus, Micromonospora sp. X14 is certainly a Carnoulès indigenous bacterium. *

However, FISH experiments could provide other informations, especially toward the proportion of each genus compared to the whole community. These informations would allow to determine if the isolated bacteria indeed belong to the “rare biosphere”.

Another concern stems from the fact that many of the isolates are highly related to those presently known, eg. 97-99% similarity. If these were in fact previously undetected low abundance community members you would expect some novelty from AMD.

***Authors’ response:****Members belonging to 3 genera, as defined by Tindall *[23]*, were isolated in this study. One cannot expect only novelty when exploring the cultured bacteria. Most of the bacteria isolated here are newly detected in Carnoulès, but this does not mean that all of them are completely new in databases. It only means that they were not detected, maybe because of molecular biases, and/or maybe because they were, in the Carnoulès conditions, not in sufficient abundance to be detected by molecular approaches. But related strains can become abundant in other environments, leading to their detection by PCR.*

The authors state in the abstract,“This work highlights that bacterial diversity in AMDs is much higher than previously envisaged”. Sure, assuming these isolates originate from the creek which is difficult to say. It possible that they are transient members of the community and likely not metabolically active.

***Authors’ response:****We agree with Reviewer 3, we cannot rule out that some of these bacteria may be “transient”, in the same manner to the possible “transient” presence of strains detected by culture-independent approaches. Many studies focused on determining the bacterial diversity in any environment in a single time point. Unless repeated studies, the observed biodiversity is a snapshot of what is present at a given time. This is true for both culture-dependent and –independent approaches. An interesting approach would be to repeat this culture-dependent study over time.*

As has been shown all environments have a tail of “rare” species, if these genera are present in the rare component of the community it likely means they are not major contributors to the ecology of the system. Generally the greatest contributions to the field come when isolates are obtained from the dominant members of the community.

***Authors’ response:****This is true in general. However, some biological functions can be carried by members of the rare biosphere, as presented in the main text (see*[31]*for a good example). In addition, the bacteria isolated here may serve as a “seed bank”, becoming important in case of environmental changes. They are therefore important for the community stability by maintaining every ecological processes.*

As stated in the conclusions the main conclusions drawn from this are:

1. “Our study provides evidences that culture-dependent and culture-independent approaches enable the characterization of a different diversity compared to the one obtained by culture-independent” - This has been shown in countless other papers from the last ~15 years and is not a new finding.

***Authors’ response:****Studies comparing both approaches often lead to the conclusion that culture-based approaches allow the detection of a much smaller diversity as compared to molecular approaches, which is not the case in our study. This is one of the novelties of our paper.*

2. “the community structure is not as simple as previously established” – Until authors are able to provide evidence that they isolates are endemic to the site this is unsubstantiated.

***Authors’ response:****We understand the Reviewers’ point of view, but the results provided evidences that our isolates come from Carnoulès, the best example being Micromonospora sp. X14.*

L19-20 – what is a “cultured compartment” of an ecosystem?

***Authors’ response:****We replaced “compartment” with “bacteria”.*

L21 – What makes you think that obtaining isolates from a environment will enable the “access to the largest bacterial diversity”.

***Authors’ response:****We added the term “cultured” in “access to the largest cultured bacterial diversity”.*

L29-30 – how is a genera “unknown in databases”, I think you meant to say they are novel?

***Authors’ response:****We replaced “unknown” with “novel”*

I found the text in Figure 1 to be too small to read.

***Authors’ response:****Unfortunately, we cannot change the size of the characters in the tree labels without having an enormous tree (due to the number of characters in some labels), making impossible to better define it. However, when enlarged on the screen, it is still possible to read the labels without problem due to pixelisation.*

**Quality of written English:** Needs some language corrections before being published

***Authors’ response:****The manuscript has been revised for corrections.*

The authors would like to thank Reviewer 3 for his comments, which enabled us to precisely explain how we worked. This discussion is even more interesting, since the research community will be able to easily see the gap, but also the complementarity, between molecular and culture approaches and the point of views of the respective researchers.

Reviewers’ response:

This study is of interest in its uniqueness of design. Honestly, upon first reading it was not clear to me why Delavat et al. used media with pH ranging from 3.5 to 9.8 when the creek ranges in pH from 2.7-3.4. They state their motivation for this was to obtain a larger bacterial diversity (and novelty) from this community containing low species richness, as well as determine possible ecological/metabolic roles of low abundance community members. Assuming these Bacteria are not too transient (just passing through), it is clear that the Bacteria they obtained are not dominant members of the community, based on prior studies. Thus, they suggest that these species may be components of the rare biosphere in the creek.

This topic is timely, as the role of the rare biosphere is of considerable interest. Are rare members primarily transient and not particularly active? To what extent do they play key ecological roles, such as keystone species? In this study, they do address possible metabolic roles of these isolates by testing for cellulose degradation and arsenic oxidation for example. However, these experiments were run in media with pH that is not representative of the environment they were recovered from. Thus, it is not likely that these organisms would be too happy do this at lower pH. Therefore, it is possible that these species are part of the “seed bank” and during seasonal disturbances they may be important in maintaining stability of the community. I know for a fact that Actinobacteria are common to other AMD sites. It would be interesting to know if these species are common to rare members of acid mine drainage, that would suggest they are stable members of the communities seed bank and are important in some capacity. They also point out that this study is unique in that they were able to obtain greater diversity by culturing than by culture-independent methods, a 70% increase. This was not that convincing to me, as previous methods (eg. clone libraries) were limited by sensitivity. I bet if they were to high-throughput (tag-sequence) 16S rRNA genes from the same sample they were uncover far more.

**Quality of written English:** Acceptable

***Reviewers’ response:****We would like to thank reviewer 3 for his comments, and apologize for the English used in our previous answers. He is fully right when he says “Thus, it is not likely that these organisms would be too happy do this at lower pH”. In fact, the strain Q8 belonging to the genus Paenibacillus (isolated in this study) was studied for its capability to degrade polymers at lower pH. We demonstrated that this strain could degrade starch and xylan under a wide pH range. However, its polymer degrading activity significantly decreased at a pH under 4 *[16].

Some of our isolates were detected for the first time in AMDs, whereas others are frequently detected in such environments, suggesting that “they are stable members of the communities seed bank and are important in some capacity” as reviewer 3 suggested. X11 is the most remarkable example, since members of this previously uncultured genus were frequently detected in AMDs and other acidic environments (“Rio Tinto” in Spain, “Lower Red Eyes” in Pennsylvania, “Wheal Jane” in England (NCBI-nr BLAST, unpublished results)). This suggests a possible role of X11 and relatives in the functioning of these ecosystems.

Again, we agree with reviewer 3 when he says: “I bet if they were to high-throughput (tag-sequence) 16S rRNA genes from the same sample they were uncover far more”. We indeed think that high-throughput methods could/will uncover some of the genera detected by culture, and this would be an interesting comparison to be done.

## Supplementary Material

Additional file 1 * 16S-specific primers used for each genus.* Click here for file
