# Supplementary material for: Novel and unexpected bacterial diversity in an arsenic-rich ecosystem revealed by culture-dependent approaches
Source: Biol Direct. 2012 Sep 10;7:28. doi: 10.1186/1745-6150-7-28 (PMC3443666; doi:10.1186/1745-6150-7-28)
Supplement: Additional file 1 — 16S-specific primers used for each genus. [file 1745-6150-7-28-S1.doc]

**Additional file 1**

X14-R

Cagggaaccggagaggc

U2-R

GGATTCCGTGGAAGG

J9-R

CGCGGAGGACGTGGAATG

Q7-R

GGAAACCGTGGAATGGTC

E10-R

CGGAACTCGTGGAATGAGC

H7-R

CGACGACGTGGAATGTCG

H7p-R

CGGAGAACGTGGAATGTCC

N3B-R

CGGCGGGATTGGGTACC

Q9-R

GCACTAAAGGGCGGAAACC

I10-R

GACACTGAGAAACTAAGTTCC

K16-R

CACTGCGTGACTAAGTCAC

L12-R

CCCGACATTACTCGCTGG

K4-R

CCCACCATTACGTGCTGG

K7-R

CCACTAAGATCTCAAAGGATC

16S-specific primers used for each genus.

X14-F

GGTTTGGTGGAAAGTTTTTCG

U2-F

GGAAAGATTTATCGGTGCAG

J9-F

GGAAAGCCTTGTGCGGTC

Q7-F

GTGTTGGAAAGATTTATCGGT

E10-F

CTGGAAAGATTTATTGGTCTG

H7-F

GGTTGAAAGCTCCGGCGG

H7p-F

GTGGGTTGGAAAGATTTTTTG

N3B-F

CGGGTCAAAGGAGCAATTC

Q9-F

GGTTTACGCTGTCACTTATAG

I10-F

GGGTCAAAGGCGCAAGTC

K16-F

GAGGTTTAAAGGCGCGAGT

L12-F

GCCTCGCGCGGTCAGAC

K4-F

GGCTCGCAAGACCTCGC

K7-F

GGATCTTCGGACCTCACG

X1-R

CCACTGACAAGCATGCTTG

N4-R

CCACTAAGCTGCAAGCAGC

Q8-R

GCACCAAGGGTATCGAAAC

X1-F

GTGCCCTTCGGGGGAAAG

N4-F

CGATCGCGAGATGAAAGATTT

Q8-F

CGGAGCAATCTGTCACTTG
